# Hormetic response to B-type procyanidin ingestion involves stress-related neuromodulation via the gut-brain axis: Preclinical and clinical observations

**DOI:** 10.3389/fnut.2022.969823

**Published:** 2022-09-07

**Authors:** Naomi Osakabe, Taiki Fushimi, Yasuyuki Fujii

**Affiliations:** ^1^Functional Control Systems, Graduate School of Engineering and Science, Shibaura Institute of Technology, Saitama, Japan; ^2^Department of Bio-Science and Engineering, Shibaura Institute of Technology, Saitama, Japan

**Keywords:** B-type procyanidin, hormesis, sympathetic nervous system (SNS), central nervous system, hemodynamics, stress

## Abstract

B-type procyanidins, a series of catechin oligomers, are among the most ingested polyphenols in the human diet. Results of meta-analyses have suggested that intake of B-type procyanidins reduces cardiovascular disease risk. Another recent focus has been on the effects of B-type procyanidins on central nervous system (CNS) function. Although long-term B-type procyanidin ingestion is linked to health benefits, a single oral intake has been reported to cause physiological alterations in circulation, metabolism, and the CNS. Comprehensive analyses of previous reports indicate an optimal mid-range dose for the hemodynamic effects of B-type procyanidins, with null responses at lower or higher doses, suggesting hormesis. Indeed, polyphenols, including B-type procyanidins, elicit hormetic responses *in vitro*, but animal and clinical studies are limited. Hormesis of hemodynamic and metabolic responses to B-type procyanidins was recently confirmed in animal studies, however, and our work has linked these effects to the CNS. Here, we evaluate the hormetic response elicited by B-type procyanidins, recontextualizing the results of intervention trials. In addition, we discuss the possibility that this hormetic response to B-type procyanidins arises via CNS neurotransmitter receptors. We have verified the direction of future research for B-type procyanidins in this review.

## Introduction

**FIGURE 1:**
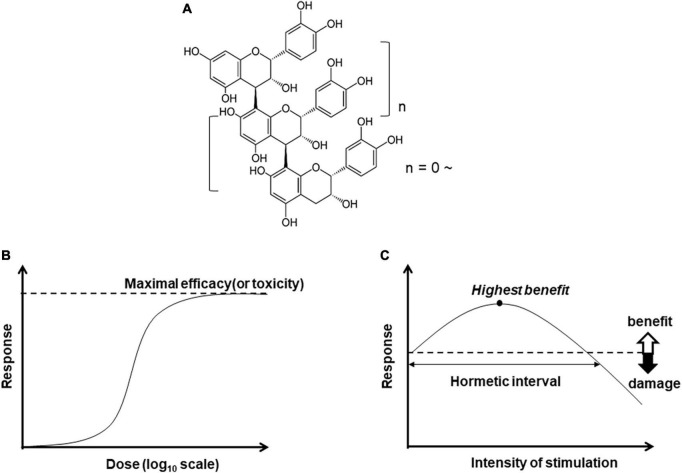
Chemical structure of B-type procyanidin (C4–C8) bond, **(A)**, and dose-response curves: **(B)** monotonic and **(C)** hormesis.

B-type procyanidins are characterized by a series of heteropolyflavan-3-ols, with a single interflavan bond between carbon-4 of the B-ring and either carbon-8 or carbon-6 of the C-ring (1–3). B-type procyanidins can be categorized by their degree of polymerization: monomers form linkages leading to oligomers. The most common monomeric unit is (–)-epicatechin, and the C4–C8 bond (Figure 1A) is the most prominent. B-type procyanidins containing 2–7 monomeric units are defined as oligoprocyanidins which are abundant in cocoa (4–6), apples (7, 8), grape seeds (9, 10), and red wine (11–13).

Results of meta-analyses have suggested that intake of foods rich in B-type procyanidins is linked to reduced risk for cardiovascular disease, including coronary heart disease, myocardial infarction, and stroke (14–20). Randomized controlled trials and subsequent meta-analyses have confirmed that dark chocolate containing large amounts of B-type procyanidins can mitigate states related to the metabolic syndrome, including hypertension (21–23), dyslipidemia (24, 25), and glucose intolerance (25, 26). In addition, the latest large-scale randomized trial found a 27% reduction in cardiovascular death by ingestion of cocoa flavanol fraction, which is rich in B-type procyanidin monomer and oligomers, for 3.6 years (27). Recent studies have focused on the benefit of B-type procyanidin ingestion for the central nervous system (CNS). A few intervention trials have reported that B-type procyanidin might be effective in improving cognitive function (28–31).

Almost all B-type procyanidins ingested in food move into the colon, and some are degraded by the microbiome (32–34). Consequently, changes in the gut microbiome induced by ingestion of B-type procyanidins for a comparatively long period may alter the composition of metabolites in the colon (32, 35–38). One hypothesis is that these colon changes associated with gut microbiota contribute to the beneficial effects of B-type procyanidins.

Acute physiological changes have been reported to follow a single intake of foods rich in B-type procyanidins. These changes are related to hemodynamics (39–43), metabolism (44, 45), the autonomic nervous system (46), and cognitive function or cerebral blood flow (28, 47–54). These findings highlight the need to evaluate the acute and chronic physiological effects of B-type procyanidin ingestion.

In addition, the acute hemodynamic changes following ingestion of B-type procyanidin, such as flow-mediated dilation (FMD), do not show a monotonic dose response (55). Instead, these physiological changes follow a pattern of hormesis, with peak benefit at mid-range doses and less benefit at higher or lower doses. Comprehensive analyses of many earlier findings suggest that there is likely a mid-range optimal dose for the effects of B-type procyanidins on hemodynamics.

Polyphenols, including B-type procyanidins, elicit hormetic responses in cell culture (56–59). Cellular proliferation occurs at relatively low concentrations, but cytotoxicity is detected at high concentrations (60). *In vivo* animal and human dose-response findings for B-type procyanidins are relatively limited. Recently, however, results from animal studies confirmed that hemodynamics and metabolism show a hormetic dose-response to B-type procyanidin, and we found that these changes arise through sympathetic nerve activation, driven by CNS activation. Here, we review data from human intervention trials supporting a hormesis pattern of response to B-type procyanidins. Furthermore, we discuss the possibility that B-type procyanidins elicit this response via neurotransmitter receptors expressed in the CNS.

### Hormesis

Bioactive compounds are expected to yield a monotonic dose-dependent response in terms of efficacy or toxicity (Figure 1B). In some cases, however, the pattern is characterized by an inverted U-shaped dose-response (Figure 1C). This pattern of hormesis can also reflect an adaptive response. For example, exposure to low amounts of a substance or stressor can induce resistance to higher doses of the same trigger. This exposure to mild levels of harmful factors can precondition a cell or an organism, inducing activation of stress resistance pathways and expanding maintenance and repair capacities (61, 62).

As an example, a moderate exercise program yields various benefits, such as decreased risk of cardiovascular disease, stronger bones and skeletal muscle, and longevity. An overly intense exercise program, however, can lead to harmful effects (63), so that the response to exercise “dose” shows a hormetic pattern. Exercise-related enhancement of cognition and mood also shows a hormetic response (64) that is reported to relate closely to adult hippocampal neurogenesis (65). Furthermore, moderate physical activity is generally accepted to be associated with cardiovascular (66–68) and metabolic benefits through sympathetic nervous system (SNS) (69–71). Recent evidence also links CNS plasticity to the effects of moderate exercise on SNS activity (72).

Polyphenols elicit a hormetic response in cell culture (73); curcumin (74), resveratrol (75) and B-type procyanidins (76). The hormesis effect of other polyphenols also has been confirmed *in vitro*, and these activities are considered to arise from modulation of a number of redox-based signaling pathways. Abundant evidence thus supports a hormesis effect of polyphenols in *in vitro* studies, but limited data illustrate these effects *in vivo*.

### Hormetic response to B-type procyanidin in intervention and animal studies

As mentioned above, repeated ingestion of B-type procyanidins is reported to reduce the risk of cardiovascular diseases. Besides numerous intervention trials have been examined following the single ingestion of foods rich in B-type procyanidins. Regarding hemodynamics, a single ingestion was associated with increased FMD at about 2 h following ingestion (23). These results indicated that a single treatment of B-type procyanidin might improve vascular endothelial cell function. Sun et al. assessed the dose-response pattern of human endothelial function to B-type procyanidin in cocoa (55). They concluded that cocoa flavanols could significantly improve endothelial function, with an optimal dose of about 710 mg. They also observed a non-linear association (inverted U-shape) between cocoa flavanols and FMD. There were no notable adverse effects in the intervention studies using 1.4 times (1,008 mg) or 1.76 times (1,248 mg) the effective dose (710 mg) shown by Sun et al. (55). Since these intervention studies used cocoa drinks or chocolate as the test food, it was limited intake amount. Therefore, the toxicity of type B-type procyanidins may not detect.

In addition, results of a recent intervention trial indicate that repeated supplements of B-type procyanidins are associated with improvement on memory tasks that depend on dentate gyrus functions (52). Intervention trials have been conducted in young adults, examining the effects on a memory task of a single ingestion of cocoa flavanols at doses from 172 to 994 mg (77). In almost all cases, cocoa flavanols were associated with enhanced working memory or mood and reduced fatigue, but evidence of dose-response in CNS studies is limited.

These results, taken together, suggest that a hormetic physiological response following a single intake of B-type procyanidins is likely. Studies of other polyphenols, such as curcumin and resveratrol, are too limited to allow for interpretations regarding dose response (78).

Repeated oral gavage with 10 mg/kg body weight (bw) of cocoa flavanol in rats resulted in significantly decreased blood pressure and markedly increased aortic endothelial nitric oxide synthase expression (eNOS), indicating this dose as optimal (79). On the other hand, a single oral administration of 10 mg/kg cocoa flavanol, resulted in a transient increase in mean blood pressure (BP) and heart rate (HR), along with a marked increase in blood flow in the cremaster muscle arteriole soon after treatment. A significant increase in eNOS phosphorylation was also observed in aorta dissected 60 min after this treatment. Similar but weaker alterations were observed at a dose of 1 mg/kg cocoa flavanol but 100 mg/kg cocoa flavanol did not trigger any changes in hemodynamics or eNOS phosphorylation.

**FIGURE 2:**
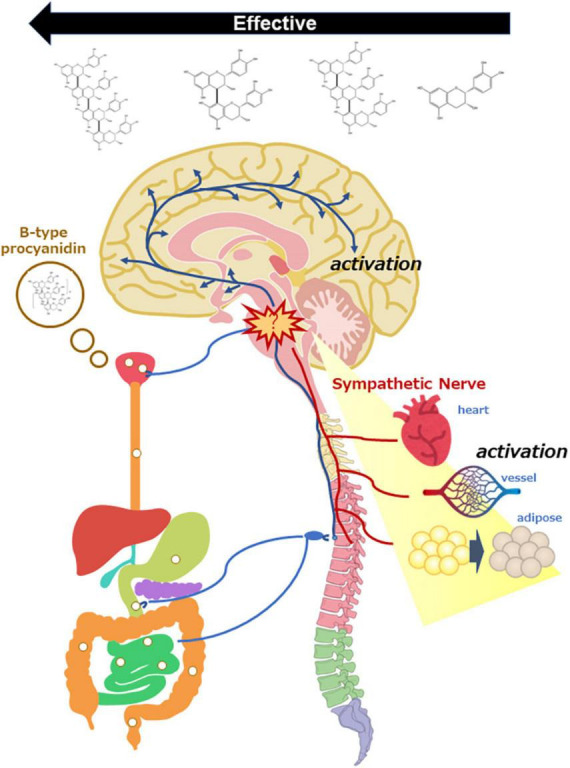
Scheme of sympathetic nerve activation by oral administration of B-type procyanidins, consequently activation of the central nervous system and peripheral organs.

We also compared B-type procyanidins such as the monomer [(-)-epicatechin; EC], dimer (procyanidin B2; B2), trimer (procyanidin C1; C1), and tetramer (cinnamtannin A2; A2) on hemodynamics (80). At a dose of 10 μg/kg, A2 and B2 were associated with a marked increase in cremasteric arteriole blood flow, C1 was linked to a slight increase, and EC did not trigger any change. Based on these findings, a relative efficacy of B-type procyanidins on hemodynamics was suggested as follows: A2 > B2 > > C1 > > > EC (Figure 2). A dose-response study of A2 showed increased blood flow with a single dose of 10 μg/kg, but not with a dose of 100 μg/kg. In our dose-response study for A2 induction of thermogenic uncoupling protein (UCP)-1 expression in brown adipose tissue (BAT), we found that a single oral dose at 1 μg/kg was associated with significantly increased UCP-1 mRNA expression (Figure 2), but more than 1 μg/kg A2 (10 to 1,000 μg/kg) did not show any change (81).

Taken together, the results of animal studies of cocoa flavanol or the B-type procyanidins are consistent with those of intervention studies following a single intake of food rich in B-type procyanidin. The implication is that this polyphenol elicits an inverted U-shaped dose-response.

### Target organ of B-type procyanidins from the perspective of bioavailability

B-type procyanidins show poor bioavailability, and intact forms in foods are hardly present in the blood (82). For this reason, how these polyphenols exert beneficial effects remains unclear. Recent studies suggest that the physiological changes following repeated B-type procyanidins ingestion may be related to alterations in gut microflora and/or their metabolites, but the mechanism for changes arising immediately after a single dose is unclear. Considering that most B-type procyanidins are present in the feces, the target organ of them is the gastrointestinal tract, including the oral cavity.

Single doses of B-type procyanidins do not draw a monotonic dose-response, and benefits are seen at the mid-range doses but not at lower or higher doses. Among various pharmacological agents, those that support social interactions or memory are reported to show biphasic reactions (83) and enhance memory (84). A single oral ingestion of cocoa flavanol has been also reported to improve cognition and mood in intervention studies. As noted, the primary target organ of B-type procyanidins appears to be the digestive tract, but activation of the CNS may be crucial to the mechanism of action.

### Sympathetic nerve activation by B-type procyanidins

**FIGURE 3:**
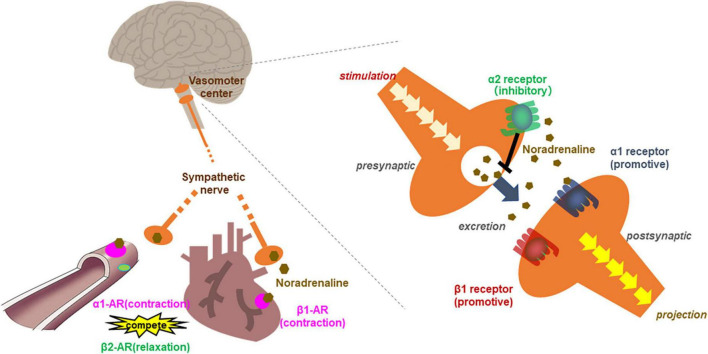
Scheme of the relationship between adrenergic receptors in the circulation system. β1 adrenergic receptors contracts the myocardium and increased cardiac output and heart rate. α1 adrenergic receptor in vascular smooth muscle contracts blood vessels, leading to elevated blood pressure. While β2 receptor relaxes vascular smooth muscle as a competitive effect against α1 adrenergic receptors. In the vasomotor center, presynaptic neurons release noradrenaline (NA) into the synaptic cleft upon stimulation. Noradrenaline binds the activating receptors of the postsynaptic neuron. NA also binds inhibitory autoreceptors of the presynaptic neurons, consequently, inhibited NA release from postsynaptic neurons.

A single oral optimal dose of cocoa flavanol triggers an increase in blood flow in the cremaster arteriole soon after treatment in rats (85). Such a rapid response likely does not depend on absorption or distribution in the blood. The SNS is a well-known regulator of hemodynamic reflection, exerting its influence through adrenergic receptors (AdR) expressed in the myocardium, vascular smooth muscle, and vasomotor center in the medulla oblongata (86). Activation of myocardial β1 AdR, which are expressed predominantly in cardiac tissue, causes increased cardiac output and HR. Activation of the α1 AdR in vascular smooth muscle contracts blood vessels, leading to elevated BP (Figure 3) (87). For this reason, we used adrenaline blockers to examine whether the SNS is involved in the hemodynamic changes induced by B-type procyanidins. We found that a transient increase in HR caused by an optimal dose of cocoa flavanol could be markedly decreased by co-treatment with a β1 AdR blocker in rats. In addition, co-treatment with an α1 blocker inhibited the transient elevation in BP that a single oral dose of cocoa flavanol induced.

Sympathetic nervous system also regulates non-shivering thermogenesis through β3 AdR in BAT via UCP-1 (88). We have found that UCP-1 mRNA upregulation in BAT after an optimal dose of cocoa flavanol is markedly attenuated by co-administration of β3 blocker. These results implicate the SNS in the acute hemodynamic and metabolic changes following a single oral dose of B-type procyanidins.

In hormesis, effects at high doses can be less than effects at optimal doses. We evaluated the hormetic pattern of response to B-type procyanidins *in vivo* and found that a single oral administration of 10-fold the optimal dose of cocoa flavanol in rats yielded no transient hemodynamic alterations (79). In addition, as noted, an optimal dose of cocoa flavanol increased UCP-1 mRNA expression in BAT, but this change was markedly dampened at doses 10-fold the optimal level (89). Based on our findings linking the hemodynamic and thermogenic effects of B-type procyanidins to SNS activation, we focused on why optimal dose elicit these effects but not high doses.

Blood pressure and heart rate are regulated competitively by inhibitory and activating AdR. Activation of the β2 receptor relaxes vascular smooth muscle as a competitive effect against the vasoconstrictive action of α1 AdR and thus decreases BP (Figure 3) (87). In our co-administration study with a high dose of B-type procyanidins and α1 blocker in rats, although, we found no changes in BP (79). Besides, inhibitory α2 AdR, which are expressed in the preganglionic sympathetic fibers and vasomotor center in the CNS, down-regulate the SNS. Yohimbine is an α2 blocker that is reported to be more effective in CNS than SNS. Given this pattern, we conducted a co-administration study with a high dose of cocoa flavanol (100 mg/kg) and yohimbine. A single high dose of cocoa flavanol alone elicited no change in BP, but BP increased markedly and transiently by co-administered with yohimbine. Similar results were observed co-administration of B-type procyanidin tetramer A2 (100 μg/kg) and yohimbine (80).

As mentioned above, whereas a single oral dose of 1 μg/kg of A2 significantly increased UCP-1 mRNA expression in BAT, doses from 10 to 1,000 μg/kg A2 did not (81). In contrast, co-administration of a high dose (100 μg/kg) of A2 and yohimbine markedly increased UCP-1 mRNA expression. A recent report suggested that the premotor neurons controlling thermogenic effector activation lie primarily within the medullary rostral raphe pallidus (90). Non-shivering thermogenesis through the β3 receptor is inhibited by α2AR activation in this region (91).

α2 AdR are present on noradrenergic terminals in the peripheral nervous system and the CNS (92). α2 adrenergic autoreceptors lie in the presynaptic membrane of adrenergic neurons, inhibiting exocytosis of their neurotransmitters (mostly noradrenaline) as part of a negative feedback loop (93, 94) (Figure 3). Feedback inhibition of noradrenaline release from sympathetic nerves by α2-autoreceptors limits its exocytosis and protects heart under normal conditions (95). The reduced hemodynamic and metabolic output at a high dose of B-type procyanidins observed in our previous studies may cause activation of autoreceptor α2. Thus, SNS deactivation may be induced by a high dose of B-type procyanidins.

### Stress and hormetic response to B-type procyanidins

The relationship between stress and hormetic responses is well known. Various factors induce the stress response, which involves rapid activation of the sympathetic–adreno–medullar (SAM) axis and the hypothalamus–pituitary–adrenal (HPA) axis (96). In the SAM, rapid physiological adaptation mediated mainly by noradrenaline results in transient responses, such as alertness, and appraisal of the situation, enabling a strategic decision. Sympathetic modulations induced by stressors rely on direct projections from the paraventricular nucleus of the hypothalamus (PVN), locus coeruleus, and rostral ventrolateral medulla to pre-ganglionic sympathetic neurons present in the dorsal intermediolateral cellular column of the spinal cord (97). As a result, noradrenaline is secreted from sympathetic nerve terminals, leading to activation of signaling pathways that evoke changes in blood vessels, glands, visceral organs, and smooth muscle. Considering the previous results following a single oral administration of B-type procyanidins, these changes may be induced by activation of the SNS.

The PVN, which also has a role in eliciting activation of the HPA, synthesizes oxytocin, vasopressin, and corticotropin-releasing hormone (CRH), depending on the target (98). CRH excreted from the PVN to the anterior pituitary induces release of adrenocorticotropic hormone, which drives the responses associated with release of cortisol (corticosterone in rodents) from the adrenal gland in the hours following stress. When blood cortisol exceeds a certain level, it exerts negative feedback on the hypothalamic release of CRH and the pituitary release of adrenocorticotropic hormone (99). Activation of these pathways results in adaptive conditions that mediate long-term memories of the experience. Therefore, HPA activation induced by optimal stress has a strong positive effect on memory, cognition, and stress resilience (100).

If the outcome following a single dose of B-type procyanidins arises as stress response, HPA activation is expected to occur at the same time as sympathetic hyperactivity. Therefore, we examined the activation of HPA following a single dose of B-type procyanidins. In mouse PVN, the optimal dose of cocoa flavanol (10 mg/kg bw) markedly upregulated CRH mRNA, as detected by *in situ* hybridization, 240 min after administration. A dose of 50 mg/kg cocoa flavanol also showed similar alterations 60 min after administration, with a significant elevation in plasma corticosterone (101). In addition, CRH mRNA in mouse PVN was increased significantly 60 min after administration of an optimal dose of A2 (10 μg/kg), and a similar change only 15 min after administration of a 10-fold oral dose of A2 (102). Few reports have described the relationship between stress intensity and the duration of response, but our results suggested that the reaction is faster with exposure to more severe stress. Taken together, these findings indicate that stimulation with an oral dose of B-type procyanidin might be a stressor for mammals, resulting in SNS activation (Figure 2).

## Conclusion

Various stressors such as radiation, reactive oxygen species, calorie restriction, temperature, chemicals, and exercise elicit hormetic responses (103). Hormesis and the underlying biochemical pathways induced by the stressors confer protection against a range of pathological or aging processes (62). In this review, we especially focused on the hormetic alterations induced by B-type procyanidins, which are electrophilic compounds that easily cause redox reactions. The relationship between CNS activation and the chemical characteristics of B-type procyanidins remains unclear and requires further clarification. B-type procyanidins or related compounds may contribute to the beneficial effects of eating fruits and vegetables through hormetic responses induced by neuromodulation.

## Author contributions

TF and YF collected the sources and drafted this manuscript. NO constructed the conception and finally approved of this manuscript. All authors contributed to the article and approved the submitted version.

## References

[1] Rue EA Rush MD van Breemen RB. Procyanidins: a comprehensive review encompassing structure elucidation via mass spectrometry. *Phytochem Rev.* (2018) 17:1–16. 10.1007/s11101-017-9507-3 29651231 PMC5891158

[2] Rauf A Imran M Abu-Izneid T Iahtisham Ul H Patel S Pan X Proanthocyanidins: a comprehensive review. *Biomed Pharmacother.* (2019) 116:108999. 10.1016/j.biopha.2019.108999 31146109

[3] Smeriglio A Barreca D Bellocco E Trombetta D. Proanthocyanidins and hydrolysable tannins: occurrence, dietary intake and pharmacological effects. *Br J Pharmacol.* (2017) 174:1244–62. 10.1111/bph.13630 27646690 PMC5429339

[4] Hatano T Miyatake H Natsume M Osakabe N Takizawa T Ito H Proanthocyanidin glycosides and related polyphenols from cacao liquor and their antioxidant effects. *Phytochemistry.* (2002) 59:749–58. 10.1016/s0031-9422(02)00051-1 11909632

[5] Natsume M Osakabe N Yamagishi M Takizawa T Nakamura T Miyatake H Analyses of polyphenols in cacao liquor, cocoa, and chocolate by normal-phase and reversed-phase HPLC. *Biosci Biotechnol Biochem.* (2000) 64:2581–7. 10.1271/bbb.64.2581 11210120

[6] Pedan V Fischer N Bernath K Huhn T Rohn S. Determination of oligomeric proanthocyanidins and their antioxidant capacity from different chocolate manufacturing stages using the NP-HPLC-online-DPPH methodology. *Food Chem.* (2017) 214:523–32. 10.1016/j.foodchem.2016.07.094 27507506

[7] Shoji T Yanagida A Kanda T. Gel permeation chromatography of anthocyanin pigments from Rose cider and red wine. *J Agric Food Chem.* (1999) 47:2885–90. 10.1021/jf981311k 10552581

[8] Shoji T Obara M Takahashi T Masumoto S Hirota H Miura T. The differences in the Flavan-3-ol and procyanidin contents of the Japanese ‘fuji’ and ‘orin’ apples using a rapid quantitative high-performance liquid chromatography method: estimation of the japanese intake of flavan-3-ols and procyanidins from apple as case study. *Foods.* (2021) 10:274. 10.3390/foods10020274 33573087 PMC7911932

[9] Muñoz-González C Criado C Pérez-Jiménez M Pozo-Bayón M. Evaluation of the effect of a grape seed tannin extract on wine ester release and perception using in vitro and in vivo instrumental and sensory approaches. *Foods.* (2021) 10:93. 10.3390/foods100100937 PMC7824827 33466484

[10] Rajakumari R Volova T Oluwafemi OS Rajesh Kumar S Thomas S Kalarikkal N. Grape seed extract-soluplus dispersion and its antioxidant activity. *Drug Dev Ind Pharm.* (2020) 46:1219–29. 10.1080/03639045.2020.1788059 32643446

[11] Carando S Teissedre PL. Catechin and procyanidin levels in French wines: contribution to dietary intake. *Basic Life Sci.* (1999) 66:725–37. 10847859 10.1007/978-1-4615-4139-4_40

[12] Bertelli AA Das DK. Grapes, wines, resveratrol, and heart health. *J Cardiovasc Pharmacol.* (2009) 54:468–76. 10.1097/FJC.0b013e3181bfaff3 19770673

[13] Merkytė V Longo E Jourdes M Jouin A Teissedre PL Boselli E. High-Performance liquid chromatography-hydrogen/deuterium exchange-high-resolution mass spectrometry partial identification of a series of tetra- and pentameric cyclic procyanidins and prodelphinidins in wine extracts. *J Agric Food Chem.* (2020) 68:3312–21. 10.1021/acs.jafc.9b06195 31930914 PMC7993638

[14] Buitrago-Lopez A Sanderson J Johnson L Warnakula S Wood A Di Angelantonio E Chocolate consumption and cardiometabolic disorders: systematic review and meta-analysis. *BMJ.* (2011) 343:d4488. 10.1136/bmj.d4488 21875885 PMC3163382

[15] Larsson SC Virtamo J Wolk A. Chocolate consumption and risk of stroke: a prospective cohort of men and meta-analysis. *Neurology.* (2012) 79:1223–9. 10.1212/WNL.0b013e31826aacfa 22933736

[16] McCullough ML Peterson JJ Patel R Jacques PF Shah R Dwyer JT. Flavonoid intake and cardiovascular disease mortality in a prospective cohort of US adults. *Am J Clin Nutr.* (2012) 95:454–64. 10.3945/ajcn.111.016634 22218162 PMC3260072

[17] Quinones M Miguel M Aleixandre A. Beneficial effects of polyphenols on cardiovascular disease. *Pharmacol Res.* (2013) 68:125–31. 10.1016/j.phrs.2012.10.018 23174266

[18] Yuan S Li X Jin Y Lu J. Chocolate consumption and risk of coronary heart disease, stroke, and diabetes: a meta-analysis of prospective studies. *Nutrients.* (2017) 9:688. 10.3390/nu9070688 28671591 PMC5537803

[19] Larsson SC Åkesson A Gigante B Wolk A. Chocolate consumption and risk of myocardial infarction: a prospective study and meta-analysis. *Heart.* (2016) 102:1017–22. 10.1136/heartjnl-2015-309203 26936339

[20] Gong F Yao S Wan J Gan X. Chocolate consumption and risk of heart failure: a meta-analysis of prospective studies. *Nutrients.* (2017) 9:402. 10.3390/nu9040402 28425931 PMC5409741

[21] Desch S Schmidt J Kobler D Sonnabend M Eitel I Sareban M Effect of cocoa products on blood pressure: systematic review and meta-analysis. *Am J Hypertens.* (2010) 23:97–103. 10.1038/ajh.2009.213 19910929

[22] Taubert D Berkels R Roesen R Klaus W. Chocolate and blood pressure in elderly individuals with isolated systolic hypertension. *JAMA.* (2003) 290:1029–30. 10.1001/jama.290.8.1029 12941673

[23] Ried K Fakler P Stocks NP. Effect of cocoa on blood pressure. *Cochrane Database Syst Rev.* (2017) 4:Cd008893. 10.1002/14651858.CD008893.pub3 28439881 PMC6478304

[24] Darand M Hajizadeh Oghaz M Hadi A Atefi M Amani R. The effect of cocoa/dark chocolate consumption on lipid profile, glycemia, and blood pressure in diabetic patients: a meta-analysis of observational studies. *Phytother Res.* (2021) 35:5487–501. 10.1002/ptr.7183 34089280

[25] Lin X Zhang I Li A Manson JE Sesso HD Wang L Cocoa flavanol intake and biomarkers for cardiometabolic health: a systematic review and meta-analysis of randomized controlled trials. *J Nutr.* (2016) 146:2325–33. 10.3945/jn.116.237644 27683874 PMC5086796

[26] Veronese N Demurtas J Celotto S Caruso MG Maggi S Bolzetta F Is chocolate consumption associated with health outcomes? An umbrella review of systematic reviews and meta-analyses. *Clin Nutr.* (2019) 38:1101–8. 10.1016/j.clnu.2018.05.019 29903472

[27] Sesso HD Manson JE Aragaki AK Rist PM Johnson LG Friedenberg G Effect of cocoa flavanol supplementation for prevention of cardiovascular disease events: the COSMOS randomized clinical trial. *Am J Clin Nutr.* (2022) 115:1490–1500. 10.1093/ajcn/nqac055 35294962 PMC9170467

[28] Desideri G Kwik-Uribe C Grassi D Necozione S Ghiadoni L Mastroiacovo D Benefits in cognitive function, blood pressure, and insulin resistance through cocoa flavanol consumption in elderly subjects with mild cognitive impairment: the Cocoa. Cognition, and Aging (CoCoA) study. *Hypertension.* (2012) 60:794–801. 10.1161/hypertensionaha.112.193060 22892813

[29] Mastroiacovo D Kwik-Uribe C Grassi D Necozione S Raffaele A Pistacchio L Cocoa flavanol consumption improves cognitive function, blood pressure control, and metabolic profile in elderly subjects: the cocoa, cognition, and aging (CoCoA) study–a randomized controlled trial. *Am J Clin Nutr.* (2015) 101:538–48. 10.3945/ajcn.114.092189 25733639 PMC4340060

[30] Brickman AM Khan UA Provenzano FA Yeung LK Suzuki W Schroeter H Enhancing dentate gyrus function with dietary flavanols improves cognition in older adults. *Nat Neurosci.* (2014) 17:1798–803. 10.1038/nn.3850 25344629 PMC4940121

[31] Sloan RP Wall M Yeung LK Feng T Feng X Provenzano F Insights into the role of diet and dietary flavanols in cognitive aging: results of a randomized controlled trial. *Sci Rep.* (2021) 11:3837. 10.1038/s41598-021-83370-2 33589674 PMC7884710

[32] Wiese S Esatbeyoglu T Winterhalter P Kruse HP Winkler S Bub A Comparative biokinetics and metabolism of pure monomeric, dimeric, and polymeric flavan-3-ols: a randomized cross-over study in humans. *Mol Nutr Food Res.* (2015) 59:610–21. 10.1002/mnfr.201400422 25546356

[33] Sorrenti V Ali S Mancin L Davinelli S Paoli A Scapagnini G. Cocoa polyphenols and gut microbiota interplay: bioavailability, prebiotic effect, and impact on human health. *Nutrients.* (2020) 12:1908. 10.3390/nu12071908 32605083 PMC7400387

[34] Gómez-Juaristi M Sarria B Martínez-López S Bravo Clemente L Mateos R. Flavanol bioavailability in two cocoa products with different phenolic content. A comparative study in humans. *Nutrients.* (2019) 11:1441. 10.3390/nu11071441 31247980 PMC6683251

[35] Urpi-Sarda M Monagas M Khan N Lamuela-Raventos RM Santos-Buelga C Sacanella E Epicatechin, procyanidins, and phenolic microbial metabolites after cocoa intake in humans and rats. *Anal Bioanal Chem.* (2009) 394:1545–56. 10.1007/s00216-009-2676-1 19333587

[36] Martin FP Montoliu I Nagy K Moco S Collino S Guy P Specific dietary preferences are linked to differing gut microbial metabolic activity in response to dark chocolate intake. *J Proteome Res.* (2012) 11:6252–63. 10.1021/pr300915z 23163751

[37] Urpi-Sarda M Monagas M Khan N Llorach R Lamuela-Raventós RM Jáuregui O Targeted metabolic profiling of phenolics in urine and plasma after regular consumption of cocoa by liquid chromatography-tandem mass spectrometry. *J Chromatogr A.* (2009) 1216:7258–67. 10.1016/j.chroma.2009.07.058 19671472

[38] Marhuenda-Muñoz M Laveriano-Santos EP Tresserra-Rimbau A Lamuela-Raventós RM Martínez-Huélamo M Vallverdú-Queralt A. Microbial phenolic metabolites: which molecules actually have an effect on human health? *Nutrients.* (2019) 11:2725. 10.3390/nu11112725 31717653 PMC6893422

[39] Farouque HM Leung M Hope SA Baldi M Schechter C Cameron JD Acute and chronic effects of flavanol-rich cocoa on vascular function in subjects with coronary artery disease: a randomized double-blind placebo-controlled study. *Clin Sci.* (2006) 111:71–80. 10.1042/cs20060048 16551272

[40] Monahan KD Feehan RP Kunselman AR Preston AG Miller DL Lott ME. Dose-dependent increases in flow-mediated dilation following acute cocoa ingestion in healthy older adults. *J Appl Physiol.* (2011) 111:1568–74. 10.1152/japplphysiol.00865.2011 21903881 PMC3233882

[41] Dower JI Geleijnse JM Kroon PA Philo M Mensink M Kromhout D Does epicatechin contribute to the acute vascular function effects of dark chocolate? A randomized, crossover study. *Mol Nutr Food Res.* (2016) 60:2379–86. 10.1002/mnfr.201600045 27329037

[42] Engler MB Engler MM Chen CY Malloy MJ Browne A Chiu EY Flavonoid-rich dark chocolate improves endothelial function and increases plasma epicatechin concentrations in healthy adults. *J Am Coll Nutr.* (2004) 23:197–204. 10.1080/07315724.2004.10719361 15190043

[43] Heiss C Dejam A Kleinbongard P Schewe T Sies H Kelm M. Vascular effects of cocoa rich in flavan-3-ols. *JAMA.* (2003) 290:1030–1. 10.1001/jama.290.8.1030 12941674

[44] Basu A Betts NM Leyva MJ Fu D Aston CE Lyons TJ. Acute cocoa supplementation increases postprandial HDL cholesterol and insulin in obese adults with type 2 diabetes after consumption of a high-fat breakfast. *J Nutr.* (2015) 145:2325–32. 10.3945/jn.115.215772 26338890 PMC4580960

[45] Naissides M Mamo JC James AP Pal S. The effect of acute red wine polyphenol consumption on postprandial lipaemia in postmenopausal women. *Atherosclerosis.* (2004) 177:401–8. 10.1016/j.atherosclerosis.2004.07.025 15530916

[46] Spaak J Merlocco AC Soleas GJ Tomlinson G Morris BL Picton P Dose-related effects of red wine and alcohol on hemodynamics, sympathetic nerve activity, and arterial diameter. *Am J Physiol Heart Circ Physiol.* (2008) 294:H605–12. 10.1152/ajpheart.01162.2007 18055508

[47] Decroix L Tonoli C Soares DD Tagougui S Heyman E Meeusen R. Acute cocoa flavanol improves cerebral oxygenation without enhancing executive function at rest or after exercise. *Appl Physiol Nutr Metab.* (2016) 41:1225–32. 10.1139/apnm-2016-0245 27849355

[48] Karabay A Saija JD Field DT Akyürek EG. The acute effects of cocoa flavanols on temporal and spatial attention. *Psychopharmacology.* (2018) 235:1497–511. 10.1007/s00213-018-4861-4 29502273 PMC5920121

[49] Scholey AB French SJ Morris PJ Kennedy DO Milne AL Haskell CF. Consumption of cocoa flavanols results in acute improvements in mood and cognitive performance during sustained mental effort. *J Psychopharmacol.* (2010) 24:1505–14. 10.1177/0269881109106923 19942640

[50] Field DT Williams CM Butler LT. Consumption of cocoa flavanols results in an acute improvement in visual and cognitive functions. *Physiol Behav.* (2011) 103:255–60. 10.1016/j.physbeh.2011.02.013 21324330

[51] Gratton G Weaver SR Burley CV Low KA Maclin EL Johns PW Dietary flavanols improve cerebral cortical oxygenation and cognition in healthy adults. *Sci Rep.* (2020) 10:19409. 10.1038/s41598-020-76160-9 33235219 PMC7687895

[52] Decroix L van Schuerbeek P Tonoli C van Cutsem J Soares DD Heyman E The effect of acute cocoa flavanol intake on the BOLD response and cognitive function in type 1 diabetes: a randomized, placebo-controlled, double-blinded cross-over pilot study. *Psychopharmacology.* (2019) 236:3421–8. 10.1007/s00213-019-05306-z 31236643

[53] Francis ST Head K Morris PG Macdonald IA. The effect of flavanol-rich cocoa on the fMRI response to a cognitive task in healthy young people. *J Cardiovasc Pharmacol.* (2006) 47(Suppl. 2):S215–20. 10.1097/00005344-200606001-00018 16794461

[54] Jackson PA Wightman EL Veasey R Forster J Khan J Saunders C A randomized, crossover study of the acute cognitive and cerebral blood flow effects of phenolic, nitrate and botanical beverages in young, healthy humans. *Nutrients.* (2020) 12:2254. 10.3390/nu12082254 32731478 PMC7468953

[55] Sun Y Zimmermann D De Castro CA Actis-Goretta L. Dose-response relationship between cocoa flavanols and human endothelial function: a systematic review and meta-analysis of randomized trials. *Food Funct.* (2019) 10:6322–30. 10.1039/c9fo01747j 31524216

[56] Tamir S Eizenberg M Somjen D Stern N Shelach R Kaye A Estrogenic and antiproliferative properties of glabridin from licorice in human breast cancer cells. *Cancer Res.* (2000) 60:5704–9. 11059763

[57] Maggiolini M Bonofiglio D Marsico S Panno ML Cenni B Picard D Estrogen receptor alpha mediates the proliferative but not the cytotoxic dose-dependent effects of two major phytoestrogens on human breast cancer cells. *Mol Pharmacol.* (2001) 60:595–602. 11502892

[58] Elattar TM Virji AS. The inhibitory effect of curcumin, genistein, quercetin and cisplatin on the growth of oral cancer cells in vitro. *Anticancer Res.* (2000) 20:1733–8. 10928101

[59] Oh SM Kim YP Chung KH. Biphasic effects of kaempferol on the estrogenicity in human breast cancer cells. *Arch Pharm Res.* (2006) 29:354–62. 10.1007/bf02968584 16756079

[60] Martel J Ojcius DM Ko YF Ke PY Wu CY Peng HH Hormetic effects of phytochemicals on health and longevity. *Trends Endocrinol Metab.* (2019) 30:335–46. 10.1016/j.tem.2019.04.001 31060881

[61] Mattson MP. Hormesis defined. *Ageing Res Rev.* (2008) 7:1–7. 10.1016/j.arr.2007.08.007 18162444 PMC2248601

[62] Gems D Partridge L. Stress-Response hormesis and aging: “that which does not kill us makes us stronger”. *Cell Metab.* (2008) 7:200–3. 10.1016/j.cmet.2008.01.001 18316025

[63] Gradari S Pallé A McGreevy KR. Fontán-Lozano Á Trejo JL. Can exercise make you smarter, happier, and have more neurons? A hormetic perspective. *Front Neurosci.* (2016) 10:93. 10.3389/fnins.2016.00093 27013955 PMC4789405

[64] Mattson MP. Energy intake and exercise as determinants of brain health and vulnerability to injury and disease. *Cell Metab.* (2012) 16:706–22. 10.1016/j.cmet.2012.08.012 23168220 PMC3518570

[65] Holmes MM Galea LA Mistlberger RE Kempermann G. Adult hippocampal neurogenesis and voluntary running activity: circadian and dose-dependent effects. *J Neurosci Res.* (2004) 76:216–22. 10.1002/jnr.20039 15048919

[66] Cechetto DF Shoemaker JK. Functional neuroanatomy of autonomic regulation. *Neuroimage.* (2009) 47:795–803. 10.1016/j.neuroimage.2009.05.024 19446637

[67] Charkoudian N Wallin BG. Sympathetic neural activity to the cardiovascular system: integrator of systemic physiology and interindividual characteristics. *Compr Physiol.* (2014) 4:825–50. 10.1002/cphy.c130038 24715570

[68] Halliwill JR Morgan BJ Charkoudian N. Peripheral chemoreflex and baroreflex interactions in cardiovascular regulation in humans. *J Physiol.* (2003) 552(Pt. 1):295–302. 10.1113/jphysiol.2003.050708 12897165 PMC2343329

[69] Fu Q Levine BD. Exercise and the autonomic nervous system. *Handb Clin Neurol.* (2013) 117:147–60. 10.1016/b978-0-444-53491-0.00013-4 24095123

[70] Brito NA Brito MN Bartness TJ. Differential sympathetic drive to adipose tissues after food deprivation, cold exposure or glucoprivation. *Am J Physiol Regul Integr Comp Physiol.* (2008) 294:R1445–52. 10.1152/ajpregu.00068.2008 18321949

[71] Labbé SM Caron A Chechi K Laplante M Lecomte R Richard D. Metabolic activity of brown, “beige,” and white adipose tissues in response to chronic adrenergic stimulation in male mice. *Am J Physiol Endocrinol Metab.* (2016) 311:E260–8. 10.1152/ajpendo.00545.2015 27143559 PMC4967144

[72] Mueller PJ. Exercise training and sympathetic nervous system activity: evidence for physical activity dependent neural plasticity. *Clin Exp Pharmacol Physiol.* (2007) 34:377–84. 10.1111/j.1440-1681.2007.04590.x 17324153

[73] Kanner J. Polyphenols by generating H2O2, affect cell redox signaling, inhibit PTPs and activate Nrf2 axis for adaptation and cell surviving: in vitro, in vivo and human health. *Antioxidants.* (2020) 9:797. 10.3390/antiox9090797 32867057 PMC7555200

[74] Concetta Scuto M Mancuso C Tomasello B Laura Ontario M Cavallaro A Frasca F Curcumin, hormesis and the nervous system. *Nutrients.* (2019) 11:2417. 10.3390/nu11102417 31658697 PMC6835324

[75] Zhao YN Li WF Li F Zhang Z Dai YD Xu AL Resveratrol improves learning and memory in normally aged mice through microRNA-CREB pathway. *Biochem Biophys Res Commun.* (2013) 435:597–602. 10.1016/j.bbrc.2013.05.025 23685142

[76] Fujii Y Suhara Y Sukikara Y Teshima T Hirota Y Yoshimura K Elucidation of the interaction between flavan-3-ols and bovine serum albumin and its effect on their in-vitro cytotoxicity. *Molecules.* (2019) 24:3667. 10.3390/molecules24203667 31614668 PMC6832702

[77] Martín MA Goya L de Pascual-Teresa S. Effect of cocoa and cocoa products on cognitive performance in young adults. *Nutrients.* (2020) 12:3691. 10.3390/nu12123691 33265948 PMC7760676

[78] Leri M Scuto M Ontario ML Calabrese V Calabrese EJ Bucciantini M Healthy effects of plant polyphenols: molecular mechanisms. *Int J Mol Sci.* (2020) 21:1250. 10.3390/ijms21041250 32070025 PMC7072974

[79] Saito A Inagawa K Ebe R Fukase S Horikoshi Y Shibata M Onset of a hypotensive effect following ingestion of flavan 3-ols involved in the activation of adrenergic receptors. *Free Radic Biol Med.* (2016) 99:584–92. 10.1016/j.freeradbiomed.2016.09.008 27616615

[80] Koizumi R Fushimi T Sato Y Fujii Y Sato H Osakabe N. Relationship between hemodynamic alteration and sympathetic nerve activation following a single oral dose of cinnamtannin A2. *Free Radic Res.* (2020) 55:491–8. 10.1080/10715762.2020.1759805 32321314

[81] Nakagawa Y Ishimura K Oya S Kamino M Fujii Y Nanba F Comparison of the sympathetic stimulatory abilities of B-type procyanidins based on induction of uncoupling protein-1 in brown adipose tissue (BAT) and increased plasma catecholamine (CA) in mice. *PLoS One.* (2018) 13:e0201203. 10.1371/journal.pone.0201203 30059510 PMC6066223

[82] Osakabe N Terao J. Possible mechanisms of postprandial physiological alterations following flavan 3-ol ingestion. *Nutr Rev.* (2018) 76:174–86. 10.1093/nutrit/nux070 29315425

[83] Calabrese EJ. An assessment of anxiolytic drug screening tests: hormetic dose responses predominate. *Crit Rev Toxicol.* (2008) 38:489–542. 10.1080/10408440802014238 18615308

[84] Calabrese EJ. Alzheimer’s disease drugs: an application of the hormetic dose-response model. *Crit Rev Toxicol.* (2008) 38:419–51. 10.1080/10408440802003991 18568864

[85] Ingawa K Aruga N Matsumura Y Shibata M Osakabe N. Alteration of the systemic and microcirculation by a single oral dose of flavan-3-ols. *PLoS One.* (2014) 9:e94853. 10.1371/journal.pone.0094853 24740211 PMC3989254

[86] Sear JW. 26-Antihypertensive drugs and vasodilators. In: Hemmings HC Egan TD editors. *Pharmacology and Physiology for Anesthesia (Second Edition).* Philadelphia: Elsevier (2019). p. 535–55.

[87] Grassi G. Assessment of sympathetic cardiovascular drive in human hypertension: achievements and perspectives. *Hypertension.* (2009) 54:690–7. 10.1161/hypertensionaha.108.119883 19720958

[88] Bartness TJ Vaughan CH Song CK. Sympathetic and sensory innervation of brown adipose tissue. *Int J Obes.* (2010) 34 Suppl. 1:S36–42. 10.1038/ijo.2010.182 20935665 PMC3999344

[89] Kamio N Suzuki T Watanabe Y Suhara Y Osakabe N. A single oral dose of flavan-3-ols enhances energy expenditure by sympathetic nerve stimulation in mice. *Free Radic Biol Med.* (2015) 91:256–63. 10.1016/j.freeradbiomed.2015.12.030 26738802

[90] Morrison SF Madden CJ Tupone D. Central control of brown adipose tissue thermogenesis. *Front Endocrinol.* (2012) 3:5. 10.3389/fendo.2012.00005 22389645 PMC3292175

[91] Madden CJ Tupone D Cano G Morrison SF. α2 Adrenergic receptor-mediated inhibition of thermogenesis. *J Neurosci.* (2013) 33:2017–28. 10.1523/jneurosci.4701-12.2013 23365239 PMC3711400

[92] Langer SZ. Presynaptic regulation of catecholamine release. *Biochem Pharmacol.* (1974) 23:1793–800. 10.1016/0006-2952(74)90187-7 4617579

[93] Starke K. Presynaptic autoreceptors in the third decade: focus on alpha2-adrenoceptors. *J Neurochem.* (2001) 78:685–93. 10.1046/j.1471-4159.2001.00484.x 11520889

[94] Trendelenburg AU Philipp M Meyer A Klebroff W Hein L Starke K. All three alpha2-adrenoceptor types serve as autoreceptors in postganglionic sympathetic neurons. *Naunyn Schmiedebergs Arch Pharmacol.* (2003) 368:504–12. 10.1007/s00210-003-0829-x 14610637

[95] Kurnik D Muszkat M Li C Sofowora GG Solus J Xie HG Variations in the alpha2A-adrenergic receptor gene and their functional effects. *Clin Pharmacol Ther.* (2006) 79:173–85. 10.1016/j.clpt.2005.10.006 16513442

[96] Godoy LD Rossignoli MT Delfino-Pereira P Garcia-Cairasco N de Lima Umeoka EHA. Comprehensive overview on stress neurobiology: basic concepts and clinical implications. *Front Behav Neurosci.* (2018) 12:127. 10.3389/fnbeh.2018.00127 30034327 PMC6043787

[97] Ulrich-Lai YM Herman JP. Neural regulation of endocrine and autonomic stress responses. *Nat Rev Neurosci.* (2009) 10:397–409. 10.1038/nrn2647 19469025 PMC4240627

[98] Sawchenko PE Brown ER Chan RK Ericsson A Li HY Roland BL The paraventricular nucleus of the hypothalamus and the functional neuroanatomy of visceromotor responses to stress. *Prog Brain Res.* (1996) 107:201–22. 10.1016/s0079-6123(08)61866-x 8782521

[99] Seeman TE Singer B Wilkinson CW Bruce M. Gender differences in age-related changes in HPA axis reactivity. *Psychoneuroendocrinology.* (2001) 26:225–40. 10.1016/S0306-4530(00)00043-3 11166486

[100] Finsterwald C Alberini CM. Stress and glucocorticoid receptor-dependent mechanisms in long-term memory: from adaptive responses to psychopathologies. *Neurobiol Learn Mem.* (2014) 112:17–29. 10.1016/j.nlm.2013.09.017 24113652 PMC3979509

[101] Fujii Y Suzuki K Hasegawa Y Nanba F Toda T Adachi T Single oral administration of flavan 3-ols induces stress responses monitored with stress hormone elevations in the plasma and paraventricular nucleus. *Neuroscience Lett.* (2018) 682:106–11. 10.1016/j.neulet.2018.06.015 29902479

[102] Fujii Y Suzuki K Adachi T Taira S Osakabe N. Corticotropin-releasing hormone is significantly upregulated in the mouse paraventricular nucleus following a single oral dose of cinnamtannin A2 as an (-)-epicatechin tetramer. *J Clin Biochem Nutr.* (2019) 65:29–33. 103 10.3164/jcbn.19-19 31379411 PMC6667379

[103] Berry R López-Martínez G. A dose of experimental hormesis: when mild stress protects and improves animal performance. *Comp Biochem Physiol A Mol Integr Physiol.* (2020) 242:110658. 10.1016/j.cbpa.2020.110658 31954863 PMC7066548

